# Supplementary material for: Boat anchoring contributes substantially to coral reef degradation in the British Virgin Islands
Source: PeerJ. 2019 May 23;7:e7010. doi: 10.7717/peerj.7010 (PMC6535217; doi:10.7717/peerj.7010)
Supplement: Table S1 — For each dependent variable, we summarize the statistical model used and the results of the tests for effects of anchoring (see Methods for further details and abbreviations used). [file peerj-07-7010-s001.docx]

## Table S1:

## Detailed results of statistical hypothesis tests.

For each dependent variable, we summarize the statistical model used and the results of the tests for effects of anchoring (see Methods for further details and abbreviations used).

| **Dependent variable** | **Model** | **Test statistics, df, and p-values** |
| --- | --- | --- |
| Observed yacht density | ANOVA | F_2,147_ = 12.8, p <0.00001 |
| Overturned scleractinian corals | K-W | Χ^2^ = 6.75, p = 0.034, Nemenyi p_H-L_ = 0.046, p_H-M_ = 0.78, and p_L-M_ = 0.625 |
| Broken scleractinian corals | K-W | Χ^2^ = 8.67, p = 0.013, Nemenyi p_H-L_ = 0.048, p_H-M_ = 0.72, and p_L-M_ = 0.71 |
| Broken gorgonians | ANOVA, square-root transformation, | F_2,14_ = 10.18, p = 0.002, LSM t_H-L_ = 4.40, p_H-L_ = 0.002, t_H-M_ = 1.83, p_H-M_ = 0.20, t_L-M_ = -0.659, p_L-M_ = 0.79 |
| Distance from land | ANOVA | F_2,20_ = 0.5, p = 0.60 |
| Distance from development | ANOVA | F_2,20_ = 0.3, p = 0.75 |
| Coral percent cover | MLE, beta distribution | a~Anchoring+Group, dAIC = 0, weight = 0.84, p_M-H_ = 0.047, p_L-H_ = 0.02, p_L-M_ = 0.89; b = 88.26, z_b_ = 2.82, p_b_ = 0.0048 |
| Sea fan percent cover | MLE, beta distribution | a~Anchoring, dAIC = 0, weight = 0.74, p_M-H_ = 0.9, p_L-H_ = 0.044, p_L-M_ = 0.049; b = 27.05, z_b_ = 2.70, p_b_ = 0.0069 |
| Coral colony size | ANOVA | F_2,14_ = 7.16, p = 0.007; LSM H-L t ratio = -2.66, pL-H = 0.046; H-M t ratio = -1.59, pM-H = 0.28; L-M t ratio = -0.089, pL-M = 0.996 |
| Coral colony density | MLE, lognormal distribution | μ~Anchoring; dAIC = 0, weight = 0.63; μ_H_ = 2.06, p_H_ <0.00001; μ_M-H_ = 0.21, p_M-H_ = 0.55; μ_L-H_ = 0.85, p_L-H_ = 0.00028; μ_L-M_ = 0.64, p_L-M_ = 0.068; σ = 0.54, p_σ_ <0.00001 |
| Coral species richness | ANOVA | F_2,14_ = 14.76, p = 0.0004; LSM t_H-L_ = -5.4, p_H-L_ = 0.0002; t_H-M_ = 0.31, p_H-M_ = 0.94; t_L-M_ = 3.43, p_L-M_ = 0.01 |
| Branching coral colony surface area | ANOVA, cube root transformation | F_2,13_ = 3.8, p = 0.05; no significant contrasts |
| Branching coral colony density | ANOVA, square-root transformation, | F_2,13_ = 3.9, p = 0.047, LSM t_H-L_ = -2.75, p_H-L_ = 0.041; t_H-M_ = -0.24, p_H-M_ = 0.97; t_L-M_ = 1.40, p_L-M_ = 0.37 |
| Mounding coral colony surface area | ANOVA | F_2,14_ = 26.83, p = 0.00002, LSM t_H-L_ = -6.70, p_H-L_ <0.0001; t_H-M_ = -2.898, p_H-M_ = 0.03; t_L-M_ = 0.899, p_L-M_ = 0.65 |
| Mounding coral colony density | MLE, lognormal distribution | μ~Anchoring, dAIC = 0.0, weight = 0.57; p_M-H_ = 0.69; p_L-H_ = 0.002; p_M-L_ = 0.10; σ = 0.53, z_σ_ = 6.93, p_σ_ <0.00001 |
| Plate coral colony density | MLE, gamma distribution | s~Anchoring and a~Group, dAIC = 0, weight = 0.93; p_M-H_ = 0.61; p_L-H_ = 0.026; p_M-L_ = 0.03; a_Average_ = 1.69 |
| Reef rugosity | MLE, lognormal distribution | μ~Anchoring+Group; dAIC = 0, df = 11, weight = 1; μ_H_ = 4.66, z_H_ = 55.36, p_H_ <0.00001; μ_M-H_ = 0.41, z_M-H_ = 2.61, p_M-H_ = 0.009; μ_L-H_ = 0.49, z_L-H_ = 4.18, p_L-H_ = 0.00003; μ_M-L_ = -0.075, z_M-L_ = -0.49, p_M-L_ = 0.63; σ = 0.27, z_σ_ = 7.07, p_σ_ <0.00001 |
| Total fish density | ANOVA | F = 6.26, p = 0.01; LSM tH-L = -3.8, pH-L = 0.005, tH-M = -1.3, pH-M = 0.4, tL-M = 1.2, pL-M = 0.48 |
| Adult fish density | ANOVA | F = 8.45, p = 0.004; LSM tH-L = -4.4, pH-L = 0.002, tH-M = -1.9, pH-M = 0.2, tL-M = 1.0, pL-M = 0.58 |
| Juvenile fish density | MLE, gamma distribution | s~Anchoring, pH=0.001, pM-H=0.2, pL-H=0.6, pM-L=0.08 |
| Fish species richness | ANOVA | F = 10.7, p = 0.002; LSM tH-L = -5.1, pH-L = 0.0004, tH-M = -0.9, pH-M = 0.6, tL-M = 2.5, pL-M = 0.06 |
| Adult scraper and excavator density | ANOVA | F = 5.03, p = 0.02; LSM t_H-L_ = -3.8, p_H-L_ = 0.006, t_H-M_ = -1.4, p_H-M_ = 0.4, t_L-M_ = 1.1, p_L-M_ = 0.5 |
| Other adult herbivorous fish density | ANOVA | F = 9.4, p = 0.003; LSM tH-L = -4.4, pH-L = 0.002, tH-M = -2.1, pH-M = 0.14, tL-M = 0.82, pL-M = 0.7 |
| Adult spongivore density | K-W | X^2^ = 12.6, p = 0.002; Nemenyi _pH-L_ = 0.003, p_H-M_ = 0.4, p_M-L_ = 0.4 |
| Benthic carnivore density | MLE, lognormal distribution | μ~Anchoring; dAIC = 0.0, df = 4, weight = 0.62; p_M-H_ = 0.03, p_L-H_ = 0.03, p_M-L_ = 0.63; σ = 0.46, z_σ_= 6.93, p_σ_ <0.00001 |
| Adult piscivorous fish density | MLE, lognormal distribution | μ~Anchoring+Group; dAIC = 0.0, df = 11, weight = 0.63; p_M-H_ = 0.012, p_L-H_ = 0.004, p_M-L_ = 0.73; σ = 0.64, z_σ_ = 6.93, p_σ_ <0.00001 |
