# Supplementary material for: Boat anchoring contributes substantially to coral reef degradation in the British Virgin Islands
Source: PeerJ. 2019 May 23;7:e7010. doi: 10.7717/peerj.7010 (PMC6535217; doi:10.7717/peerj.7010)
Supplement: Table S2 — Mean coral colony surface area (±95% CI) and colony density (±95% CI) at sites with low (L), medium (H) and high (H) levels of anchoring. Corals are grouped by morphological type. Proportional change is the proportional change between high and low levels of anchoring, and the last column indicates which sites are significantly different from each other using a multiple comparison test (see Methods for further details). [file peerj-07-7010-s002.docx]

## Table S2:

## Effects of anchoring on coral colony density and colony size.

Mean coral colony surface area (± 95% CI) and colony density (± 95% CI) at sites with low (L), medium (H) and high (H) levels of anchoring. Corals are grouped by morphological type. Proportional change is the proportional change between high and low levels of anchoring, and . the last column indicates which sites are significantly different from each other using a multiple comparison test (see Methods for further details).

|  | **Colony surface area** | | | | |
| --- | --- | --- | --- | --- | --- |
|  | **Low anchoring sites (cm^2^)** | **Medium anchoring sites (cm^2^)** | **High anchoring sites (cm^2^)** | **Proportional change in density (H-L) /L** | **Sites that differed** |
| **Coral morphology** |  |  |  |  |  |
| Brain | 540.3 ± 658.9 | 476.5 ± 482.2 | 143.8 ± 160.2 | -0.73 | None |
| Branching | 266.7 ± 367.9 | 50.5 ± 41.6 | 92.2 ± 115.8 | -0.65 | None |
| Cup | 28.9 ± 40.0 | 20.1 ± 34.8 | 6.1 ± 16.5 | -0.79 | None |
| Encrusting | 90.5 ± 124.4 | 147.4 ± 152.2 | 51.3 ± 87.2 | -0.43 | None |
| Mound | 295.7 ± 16.3 | 260.0 ± 36.9 | 143.4 ± 16.7 | -0.52 | H-L, H-M |
| Plate | 52.4 ± 46.4 | 60.8 ± 55.9 | 30.4 ± 19.9 | -0.42 | None |
|  |  |  |  |  |  |
|  | **Colony density** | | | | |
|  | **Low anchoring sites (#/45m^2^)** | **Medium anchoring sites (#/45m^2^)** | **High anchoring sites (#/45m^2^)** | **Proportional change in density (H-L) /L** | **Sites that differed** |
| **Coral morphology** |  |  |  |  |  |
| Brain | 0.24 ± 0.22 | 0.34 ± 0.12 | 0.12 ± 0.14 | -0.49 | None |
| Branching | 3.0 ± 1.8 | 1.4 ± 1.9 | 1.0 ± 1.0 | -0.67 | H-L |
| Cup | 0.24 ± 0.44 | 0.23 ± 0.39 | 0.04 ± 0.09 | -0.83 | None |
| Encrusting | 0.27 ± 0.3 | 0.22 ± 0.2 | 0.25 ± 0.3 | -0.07 | None |
| Mound | 12.0 ± 6.3 | 9.0 ± 8.5 | 5.8 ± 2.6 | -0.52 | H-L |
| Plate | 5.6 ± 4.3 | 2 ± 1.5 | 2.5 ± 2.0 | -0.55 | H-L, M-L |
