# Supplementary figures and images for: Boat anchoring contributes substantially to coral reef degradation in the British Virgin Islands
Source: PeerJ. 2019 May 23;7:e7010. doi: 10.7717/peerj.7010 (PMC6535217; doi:10.7717/peerj.7010)

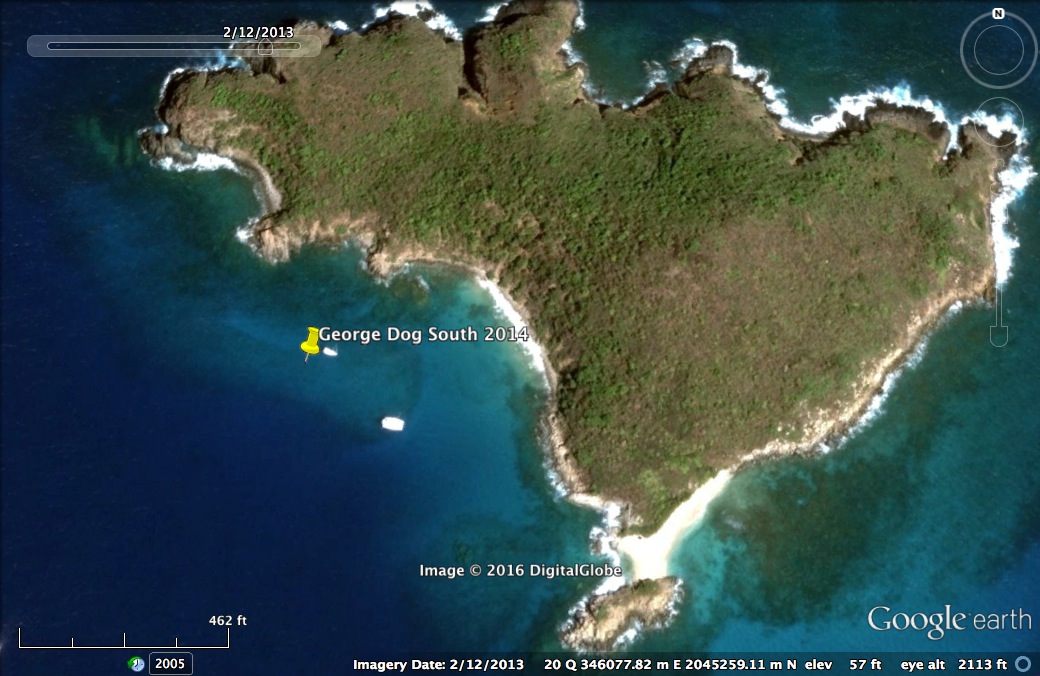

Supplement: Figure S1 — The yellow pin represents a mooring buoy, so this image has 1 moored vessel and 1 anchored one. Map Data from GoogleEarth and DigitalGlobe 2013. [file peerj-07-7010-s003.jpg]

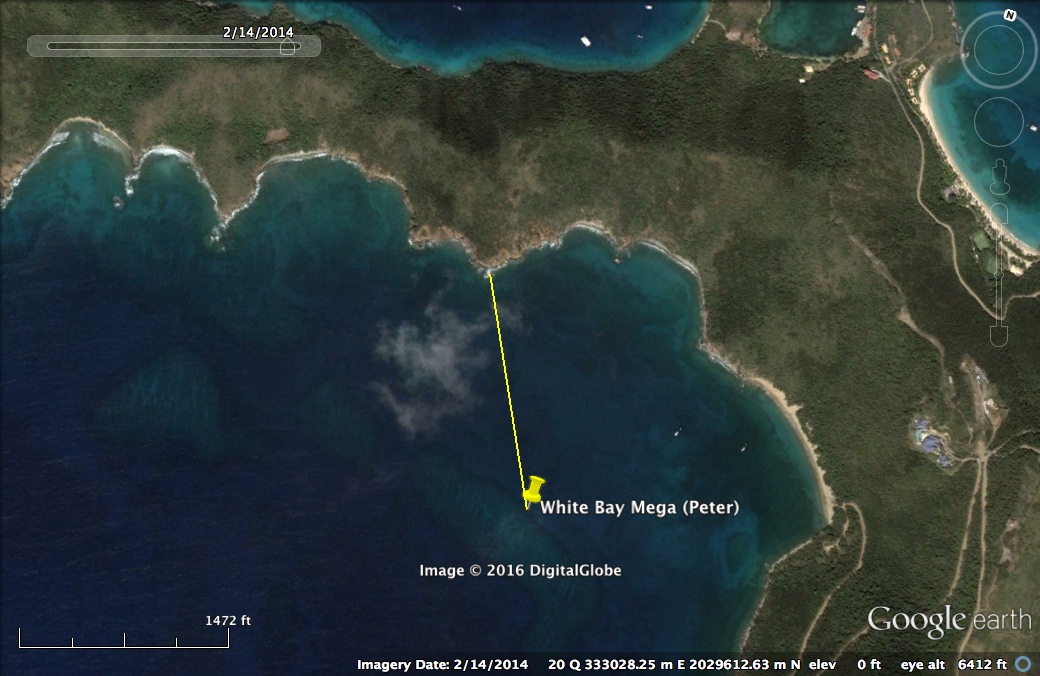

Supplement: Figure S2 — The study site indicated by the yellow pin (White Bay Mega-Peter) is a highly anchored site in White Bay near Peter Island. The line is the shortest distance to shore (0.51 km). Similar measurements were made to the nearest shore and the nearest development for all sites. Map Data from GoogleEarth and DigitalGlobe 2014. [file peerj-07-7010-s004.jpg]

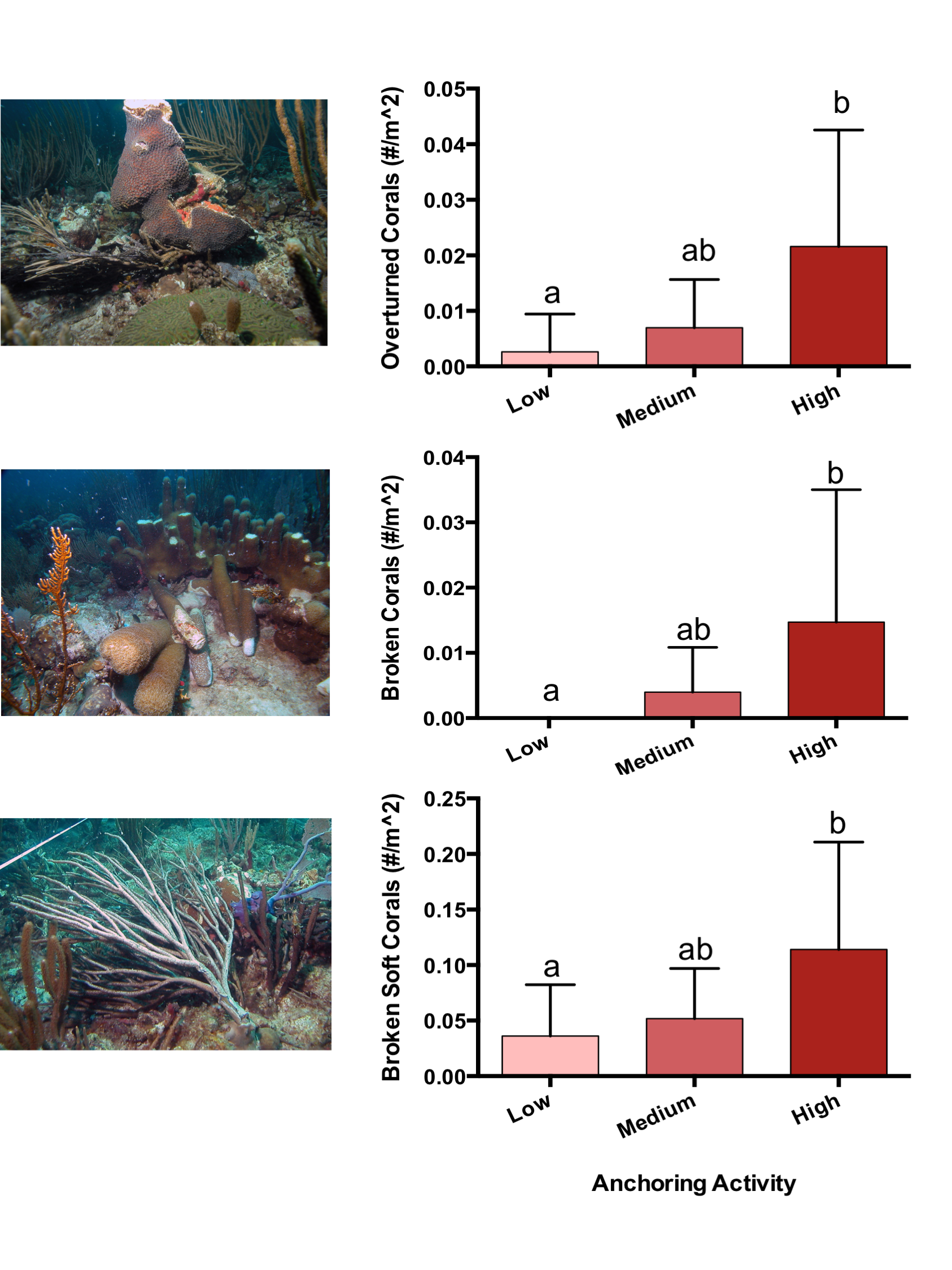

Supplement: Figure S3 — Plotted are means (±SE) of anchor damage symptoms: the density of overturned scleractinian corals (top), the density of overturned scleractinian corals (middle), and the density of broken soft corals (bottom). Letters above bars indicate significant differences based on a multiple comparison test (means that do not differ share a letter). Next to each plot is a photograph showing a typical exactly of each symptom. Photographs taken by Graham E Forrester. [file peerj-07-7010-s005.png]

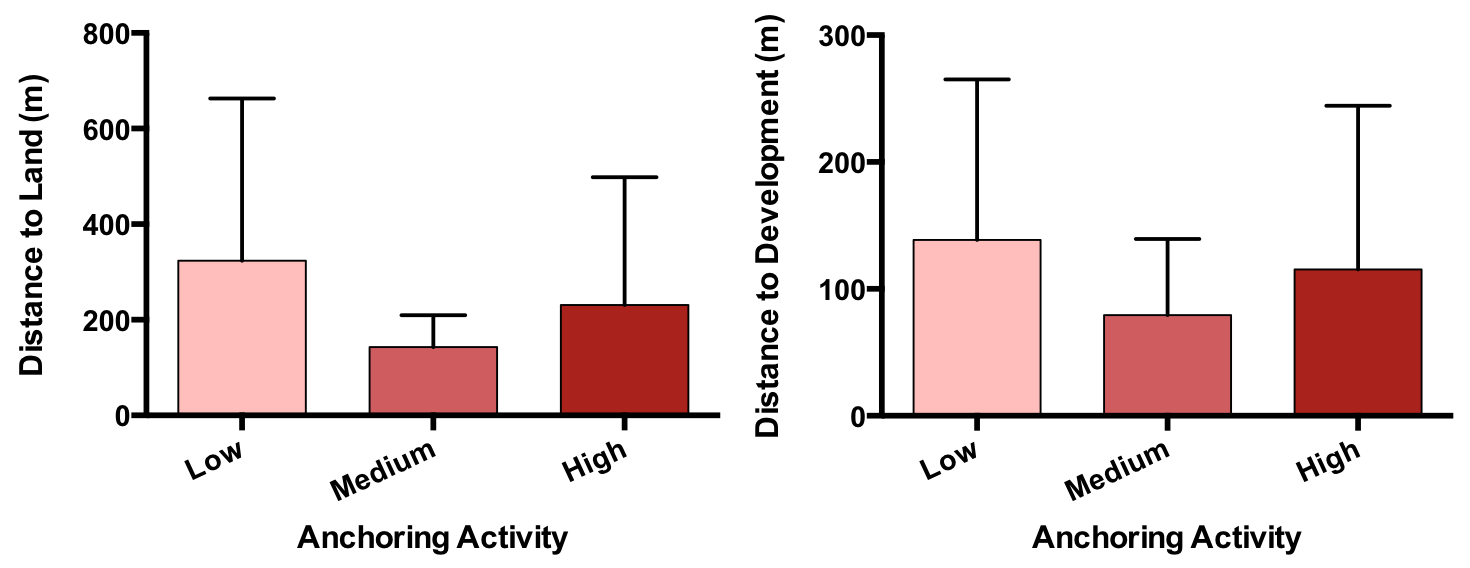

Supplement: Figure S4 — Plotted are mean (±SE) distance to shore from each site (left plot) and distance to the nearest developed site on shore (right plot). [file peerj-07-7010-s006.png]
