# Supplementary material for: Boat anchoring contributes substantially to coral reef degradation in the British Virgin Islands
Source: PeerJ. 2019 May 23;7:e7010. doi: 10.7717/peerj.7010 (PMC6535217; doi:10.7717/peerj.7010)
Supplement: Supplemental Information 2 — Contains separate sections for each section in the raw data file. [file peerj-07-7010-s008.docx]

Metadata for “Boat anchoring contributes substantially to coral reef degradation in the British Virgin Islands”

Authors: Rebecca L Flynn, Graham E Forrester

Data collection methodology: See Methods section of paper for descriptions of and references to procedures used in collecting data.

All data used for analysis for this revised manuscript are located in the Microsoft Excel Workbook called Flynn_Forrester_DataFiles.xlsx. Below are descriptions of what each tab (separate spreadsheet) contains.

AnchHist

This spreadsheet contains the data collected from GoogleEarth images of each site to assess the history of anchoring at each location. The column titles below indicate what is in each:

*Site*: The name assigned to each location surveyed.

*Group*: The number assigned to each based on proximity and similarity of characteristics for pairwise comparisons

*Anchoring*: The level of anchoring intensity (or frequency of anchoring): high, medium, or low

*Moorings*: y (yes) or n (no) for presence of mooring buoys

*Date*: the date the image was acquired by satellite

*N_Boats*: the number of boats counted in the image near the site

*N_Boats_wom*: a conservative estimate of the number of boats that are not likely moored (wom=”without mooring”).

AnchLandDev

This spreadsheet includes the data used to assess the possibly confounding variables of proximity of reefs to land and to development. This distance was measured using the shortest straight line distance from the center of the reef to the shore (land) or development.

The column titles below indicate what is in each:

*Site*: The name assigned to each location surveyed.

*Group*: The number assigned to each based on proximity and similarity of characteristics for pairwise comparisons

*Anchoring*: The level of anchoring intensity (or frequency of anchoring): high, medium, or low

*Moorings*: y (yes) or n (no) for presence of mooring buoys

*D_Land*: the distance in kilometers to the nearest shoreline from the center of the reef

*D_Dev*: the distance in kilometers to the nearest area of man-made structures/development.

Cover

This spreadsheet includes the average percent cover of each benthic cover classification category assessed. The data were collected using a point-intercept method along several transects per location and then the values for those transects were averaged for the location. The column headings indicate the following:

*Location*: The reef site assessed

*Group*: The number assigned to each based on proximity and similarity of characteristics for pairwise comparisons

*Anchoring*: The level of anchoring intensity (or frequency of anchoring): high, medium, or low

*Moorings*: y (yes) or n (no) for presence of mooring buoys

*HardCoral_Avg*: The values represent the average percent cover of hard (or stony) corals at each location

*FireCoral_Avg*: The values represent the average percent cover of fire coral at each location

*SeaFan_Avg*: The values represent the average percent cover of sea fans at each location

*SoftBranch_Avg*: The values represent the average percent cover of soft branching corals at each location

*SPErect_Avg*: The values represent the average percent cover of erect sponges at each location

*SPEncrusting_Avg*: The values represent the average percent cover of encrusting sponges at each location

*SPAll_Avg*: The values represent the average percent cover of all sponges at each location

*ALFleshy_Avg*: The values represent the average percent cover of fleshy algae at each location

*ALCalc_Avg*: The values represent the average percent cover of calcareous algae at each location

*ALCrust_Avg:* The values represent the average percent cover of crustose algae at each location

*ALFila_Avg*: The values represent the average percent cover of filamentous algae at each location

*ALAll_Avg*: The values represent the average percent cover of all algae at each location

*DeadCoral_Avg*: The values represent the average percent cover of dead coral at each location

*Rubble_Avg*: The values represent the average percent cover of coral rubble at each location

*Sand_Avg*: The values represent the average percent cover of sandy bottom at each location

*Other_Avg*: The values represent the average percent cover of anything else that did not fall into the above categories at each location

Morph

This spreadsheet contains the data used in assessing the differential impacts associated with anchoring on various morphological types of hard coral. These morphological categories were made based on growth form. The columns contain the following information:

*Date*: The date the reef was visited and assessed. In some cases the date is filled in with “All”. Some sites were visited on multiple days. For every site, the values were averaged for all corals of a particular morphology that were measured at that site.

*Site*: The name assigned to each location surveyed.

*Group*: The number assigned to each based on proximity and similarity of characteristics for pairwise comparisons

*Anchoring*: The level of anchoring intensity (or frequency of anchoring): high, medium, or low

*Moorings*: y (yes) or n (no) for presence of mooring buoys

*TotalTranL*: The total length of all transects along which corals intersecting the tape were measured.

*MorphoGrp*: morphological group or growth form, categorized as brain, branching, cup, encrusting, mound, pillar, or plate forms

*AvgL*: average length (cm) of live coral, measured perpendicular to the transect tape

*AvgW:* average width (cm) of live coral, measured parallel to the transect tape

*AvgLiveArea*: the average area of live coral, calculated as length * width

*Sum1/L_m*: The sum of the inverse of all length measurements (in meters) of all corals in each morphological group at each site.

*Strng_Val*: The “Strong Value” was calculated as the “Sum1/L_m” value for each transect divided by the transect length and then averaged across all transects per site.

*NperTran*: the average number of corals categorized as the given morphological form per transect at each site

Sites

This spreadsheet summarizes much of the hard coral data for each site. The columns contain the following information:

*Site*: The name assigned to each location surveyed.

*Group*: The number assigned to each based on proximity and similarity of characteristics for pairwise comparisons

*Anchoring*: The level of anchoring intensity (or frequency of anchoring): high, medium, or low

*Anch_val*: another way to classify the level of anchoring substituting single letters for each of the categories for ease of statistical processing. “c”=high, “b”=medium, and “a”=low. This column and the previous one are interchangeable.

*Moorings*: y (yes) or n (no) for presence of mooring buoys

*Total_Tran_D*: Total transect length in meters surveyed for a given site

*AvgN_Corals*: the average number of corals per transect (intersecting the transect tape)

*AvgN_Sp*: the average number of species per transect (intersecting the transect tape)

*AvgStrngVal:* the average “Strong Value” for the site. The “Strong Value” was calculated as the “Sum1/L_m” value for each transect divided by the transect length and then averaged across all transects per site.

*sum(1/L_m)*: The sum of the inverse of all length measurements (in meters) of all corals at each site.

*StrngVal*: the same as “AvgStrngVal” above

*AvgL*: the average length of corals as measured perpendicular to the transect tape

*AvgW*: the average width of corals as measured parallel to the transect tape

*AvgLiveArea*: the average area of living coral, calculated as length multiplied by the width of each coral and then averaged across each transect and site.

Damage

This spreadsheet shows the damage symptoms documented per transect length surveyed at each site. For each damage symptom, the values were calculated as the sum of all occurrences documented across each transect for the site divided by the total transect length surveyed. All damage symptoms within a 1m wide belt transect were counted and classified. The columns contain the following information:

*Site*: The name assigned to each location surveyed.

*Group*: The number assigned to each based on proximity and similarity of characteristics for pairwise comparisons

*Anchoring*: The level of anchoring intensity (or frequency of anchoring): high, medium, or low

*Moorings*: y (yes) or n (no) for presence of mooring buoys.

*Over_HC*: overturned hard coral

*Broken_HC*: broken or bent hard coral

*Broken_SC*: broken or bent soft coral

*Over_SC*: overturned soft coral

*Broken_SeaFan*: broken sea fan

*Broken_FC*: broken fire coral

*AllDamage*: the sum of all occurrences of all types of damage at each site divided by the total transect length surveyed

Fish

The data in the spreadsheet was used to assess anchoring-associated impacts to fish populations. Some columns of data in this spreadsheet were not used for this particular project. All numbers of fish are averages from all the transects surveyed at each site.

*RF_Site*: Name provided to the location by Rebecca Flynn for this particular study

*Island*: island most closely located to the reef site

*Anchoring*: The level of anchoring intensity (or frequency of anchoring): high, medium, or low

*Moorings*: y (yes) or n (no) for presence of mooring buoys. Instead of only indicating yes or no, this column includes some notes such as how old moorings are or how close to where we conducted our surveys they were located.

*Lionfish_removal*: level of effort by divers and others to remove lionfish from this location: low, high, or “?” for unknown.

*Group*: The number assigned to each based on proximity and similarity of characteristics for pairwise comparisons

*GF_Site*: Name provided to the location by Graham Forrester. Some of these names match with the RF_Site, however some are named so that they match what name is used in a long-term monitoring study for consistency.

*totnofish*: total number of fish

*totadfish*: total number of adult fish

*totjuvfish*: total number of juvenile fish

*plankta*: adult planktivorous fish

*planktj*: juvenile planktivorous fish

*herbdamsa_not3spot*: adult herbivorous damselfish, not including threespot damselfish (*Stegastes planifrons*)

*adrovherba*: adult roving herbivores

*rovherbj*: juvenile roving herbivores

*totherba*: all adult herbivores

*pisciva*: adult piscivores

*spongea*: adult spongivores

*scrapera*: adult scrapers

*bencarn1*: grouping of benthic carnivores

*bencarn2*: grouping of benthic carnivores

*bencarn*: all benthic carnivores

*omniva*: adult omnivores

*Njuvenilefishspecies*: number of juvenile fish species present

*Nfishspeciesfortmula*: number of fish species present

*Nadultfishspecies*: number of adult fish species present

Rugosity

This spreadsheet contains the data used in the final analysis of the three-dimensional structural complexity (or rugosity) of the reefs. All values are averages from all transects completed on each reef.

*Date*: date survey was completed

*Location*: The name assigned to each location surveyed.

*Group*: The number assigned to each based on proximity and similarity of characteristics for pairwise comparisons

*Anchoring*: The level of anchoring intensity (or frequency of anchoring): high, medium, or low

*Anch_val*: another way to classify the level of anchoring substituting single letters for each of the categories for ease of statistical processing. “c”=high, “b”=medium, and “a”=low. This column and the previous one are interchangeable.

*Moorings*: y (yes) or n (no) for presence of mooring buoys

*A_sumsqd*: the average of the sums of squares of the distances between each consecutive point at which height of reef above benthos was measured.

*A_sqrtsumsqd*: the average of the square root of the sums of squares of those distances

*trans*: a transformation taking the square root of the values in A_sqrtsumsqd.
